# Integrative identification of hub genes in development of atrial fibrillation related stroke

**DOI:** 10.1371/journal.pone.0283617

**Published:** 2023-03-23

**Authors:** Kai Huang, Xi Fan, Yuwen Jiang, Sheng Jin, Jiechun Huang, Liewen Pang, Yiqing Wang, Yuming Wu, Xiaotian Sun

**Affiliations:** 1 Department of Cardiothoracic Surgery, Huashan Hospital of Fudan University, Shanghai, China; 2 Department of Physiology, Hebei Medical University, Shijiazhuang, China; UCSD: University of California San Diego, UNITED STATES

## Abstract

**Background:**

As the most common arrhythmia, atrial fibrillation (AF) is associated with a significantly increased risk of stroke, which causes high disability and mortality. To date, the underlying mechanism of stroke occurring after AF remains unclear. Herein, we studied hub genes and regulatory pathways involved in AF and secondary stroke and aimed to reveal biomarkers and therapeutic targets of AF-related stroke.

**Methods:**

The GSE79768 and GSE58294 datasets were used to analyze AF- and stroke-related differentially expressed genes (DEGs) to obtain a DEG1 dataset. Weighted correlation network analysis (WGCNA) was used to identify modules associated with AF-related stroke in GSE66724 (DEG2). DEG1 and DEG2 were merged, and hub genes were identified based on protein–protein interaction networks. Gene Ontology terms were used to analyze the enriched pathways. The GSE129409 and GSE70887 were applied to construct a circRNA-miRNA-mRNA network in AF-related stroke. Hub genes were verified in patients using quantitative real-time polymerase chain reaction (qRT-PCR).

**Results:**

We identified 3,132 DEGs in blood samples and 253 DEGs in left atrial specimens. Co-expressed hub genes of *EIF4E3*, *ZNF595*, *ZNF700*, *MATR3*, *ACKR4*, *ANXA3*, *SEPSECS-AS1*, and *RNF166* were significantly associated with AF-related stroke. The *hsa_circ_0018657/hsa-miR-198/EIF4E3* pathway was explored as the regulating axis in AF-related stroke. The qRT-PCR results were consistent with the bioinformatic analysis.

**Conclusions:**

Hub genes *EIF4E3*, *ZNF595*, *ZNF700*, *MATR3*, *ACKR4*, *ANXA3*, *SEPSECS-AS1*, and *RNF166* have potential as novel biomarkers and therapeutic targets in AF-related stroke. The *hsa_circ_0018657/hsa-miR-198/EIF4E3* axis could play an important role regulating the development of AF-related stroke.

## Background

Cardioembolic stroke often results in severe neurological deficits and disability [1]. One of its most common causes is atrial fibrillation (AF). AF increases the overall risk of cerebrovascular events by three- to fivefold, causing one-third of all ischemic strokes [2,3]. AF patients suffering from cardioembolic strokes significantly display worse outcomes than those not suffering from them [4]. The prevalence of AF varies from 0.1% to 4% in different countries [5], causing global increases in stroke and associated disabilities and mortalities [6]. According to the Oxford Vascular Study (OXVASC) trial, AF patients aged over 80 years display a threefold increase in the risk of ischemic stroke despite the introduction of anticoagulants [7]. Although traditional stroke risk scores are widely used in clinical practice, identifying the relationship between AF and secondary stroke and predicting stroke risk in AF patients are still in high demand.

With the development of novel technologies, including microarray and next generation sequencing, the exploration of underlying molecular mechanisms behind AF-related disease becomes possible. Xie, Y et al. reported higher level of miR-641 and miR-30e-5p in serum exosomes in AF related stroke patients than in AF patients [8]. What’s more, the addition of miR-107 and miR-146a-5p to the MACE score increased the predictive performance of AF related stroke [9]. Zou, R et al. found that AF and stroke are related and ZNF566, PDZK1IP1, ZFHX3, and PITX2 genes are significantly associated with novel biomarkers involved in AF-related stroke [10]. By using GWAS analysis, Hsieh, C. S et al first identified deletions in chromosomal regions 1p36.32-1p36.33, 5p15.33, 8q24.3 and 19p13.3 and amplifications in 14q11.2 that were significantly associated with AF-related stroke and GNB1, PRKCZ, and GNG7 genes related to the alpha-adrenergic receptor signaling pathway that play a major role in determining the risk of an AF-related stroke [11].

However, no research has been performed to clarify the hub genes and underlying circRNA-miRNA-mRNA regulatory networks in AF-related stroke.

Serum miR-106 and MYL4 levels are closely related to the prevalence of atrial fibrillation, which can reflect the risk of thromboembolism in patients with atrial fibrillation and can be used as a biological indicator to predict the prognosis of patients with atrial fibrillation.

In this study, we aimed to identify AF-related differentially expressed genes (DEGs) and cardiogenic stroke-related DEGs. The DEGs of AF-related stroke was further identified, and the key modules and hub genes were explored. Finally, we identified differentially expressed miRNA (DEMis) and differentially expressed circRNA (DECircs) and constructed a circRNA-miRNA-mRNA network to elucidate the molecular mechanisms of AF-related stroke.

## Methods and materials

### Data acquisition

**Fig 1:**
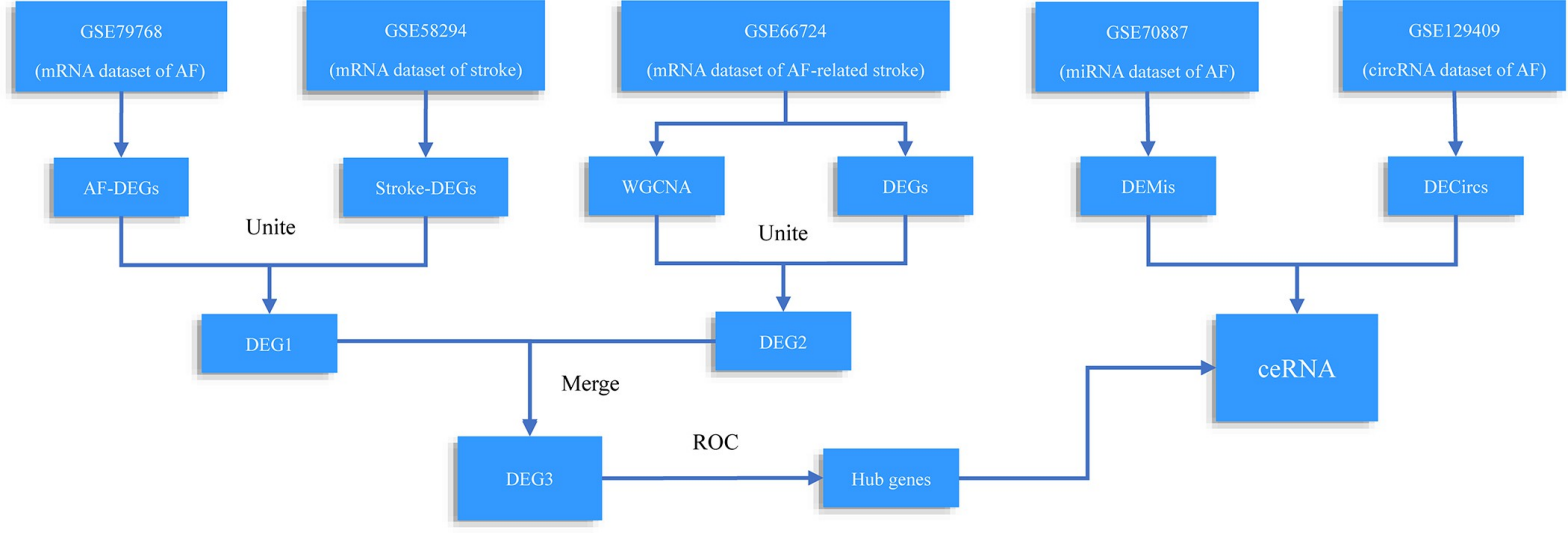
Flow chart of data preparation, processing, and analysis. GSE79768 (atrial fibrillation mRNA dataset), GSE58294 (stroke mRNA dataset), GSE66724 (atrial fibrillation related stroke mRNA dataset), GSE70887 (atrial fibrillation miRNA dataset), GSE129409 (atrial fibrillation circRNA dataset). DEMis (differently expressed miRNAs), DECircs (differently expressed circRNAs), ceRNA (competitive endogenous RNA).

Microarray datasets of stroke and AF patients were downloaded from Gene Expression Omnibus (https://www.ncbi.nlm.nih.gov/geoprofiles). The mRNA expression profile GSE79768 was performed on Platform GPL570 with paired left atrial (LA) and right atrial specimens obtained from patients with persistent AF (n = 13) or sinus rhythm (n = 13), which described the mechanisms of AF-related LA remodeling, arrhythmogenesis, and thrombogenesis. The mRNA expression profile GSE58294 was performed on Platform GPL570 containing plasma samples from patients with cardioembolic stroke (n = 69) and healthy controls (n = 23). Subjects were analyzed at three time points: less than 3 h, 5 h, and 24 h after stroke. The mRNA expression profile data GSE66724 was based on peripheral blood cells in eight patients with AF and stroke and eight AF subjects without stroke (same anticoagulation and arrhythmia therapy). The miRNA dataset GSE70887 was performed on Platform GPL19546, containing LA biopsies of two sinus rhythm and four persistent AF patients. The circRNA dataset GSE129409 was performed on GPL21825 with heart tissues from three AF patients and three healthy controls. The flowchart of this study is shown in **Fig 1**.

### Data screening strategy

The limma package in R [12] was applied to evaluate all the five matrix datasets. The Bayesian method was used to correct for batch effects. If more than one probe mapped to the same gene, the average expression value was used to equal its expression value. The Benjamini-Hochberg method was used to adjust original p-values, and the false discovery rate procedure was used to calculate fold changes (FC). The DEGs were screened by *P* < 0.05 and log FC > average (abs(log FC)) + 2 * SD (abs(log FC)) [13]. The expression values of the |log FC| > 0.618 and adjusted *P* < 0.05 were used for filtering AF-DEGs. The |log FC| > 0.62 and *P* < 0.05 were used to identify stroke-DEGs. The |log FC| > 0.273 and *P* < 0.05 were used to identify AF-related stroke-DEGs. As per DEMis and DECircs, the cutoff values for log FC were 1.14 and 2.09, respectively. Volcano maps and heatmaps were also drawn by the plot and pheatmap package in the R studio to exhibit all DEGs.

### Construction of a weighted gene co-expression network and identification of significant modules

The co-expression network of DEGs was constructed based on the GSE66724 microarray dataset using the R package “weighted correlation network analysis (WGCNA) [14].” The soft-thresholding power was set to 5 when 0.85 was used as the correlation coefficient threshold, and 50 was selected as the minimum number of genes in the modules. To merge possibly similar modules, we defined 0.25 as the threshold for cut height.

### Identification of hub genes and efficacy evaluation

DEG1 was confirmed by intersecting AF-DEGs and stroke-DEGs. Then, genes in the module of interest by WGCNA were intersected with AF-related stroke-DEGs, and DEG2 was obtained. Thereafter, DEG1 and DEG2 were merged to obtain AF-related stroke genes (DEG3). The venn plot of these three DEG datasets were showed in **S1 Fig. The PPI network of DEG3 were showed in S2 Fig.** The ROC curve was then plotted and the AUC was calculated to evaluate the capability of selected genes to distinguish AF-related stroke patients and AF patients without stroke. Only genes with an AUC > 0.8 were considered hub genes. The comparative toxicogenomic database (http://ctdbase.org/) was used to analyze possible relationships between hub genes and nervous or cardiovascular diseases (**Fig 1**).

### Functional enrichment analysis

Functional annotation of Gene Ontology (GO) [15,16] was performed with the ClusterProfiler package [17] in R studio. GO terms (biological processes, molecular functions, and cellular components) with a *P <* 0.05 were considered significantly enriched. Subsequently, the AmiGO database (v2.0; http://amigogeneontology.org/amigo/) was used to analyze the GO consortium for filtered hub genes, verify their accuracy, and annotate their biological functions.

### Protein–protein interaction (PPI) networks

To calculate the interactions between molecules in AF-DEGs, stroke-DEGs, and WGCNA modules, the online database STRING [18] (https://string-db.org/) was applied with a confidence score > 0.4. Cytoscape software (Version 3.7.2; http://cytoscape.org/) was used to visualize and analyze the biological networks and node degrees [19].

### Construction of circRNA-miRNA-mRNA network for AF-related stroke

Three online databases, including miRDB (http://www.mirdb.org), miRTarBase (http://mirtarbase.mbc.nctu.edu.tw), and TargetScan (http://www.targetscan.org), were used to predict the targets of DEMis. The targeted genes fulfilling the qualification of at least two databases were selected. Predicted genes were further filtered by matching the hub genes selected above. Then, the miRNA-mRNA pairs were determined. The miRNA targets of DECircs were predicted using the online database circBank (http://www.circbank.cn/), and they were validated by the DEMis selected above. Then, the circRNA-miRNA pairs were determined. Cytoscape was used to visualize this circRNA-miRNA-mRNA network.

### Blood sample collection and quantitative real-time polymerase chain reaction

AF patients with or without stroke in the Huashan Hospital were included in this study. The exclusion criteria were coronary artery heart disease, hypertension, diabetes, and obstructive sleep apnea syndrome. Blood samples from three AF patients with stroke and three AF patients without stroke were collected. Samples were immediately preserved in RNALock Reagent (TIANGEN, Beijing, China) to inhibit RNA degradation. This study was approved by the Medical Ethics Committee of Huashan Hospital, Fudan University. All patients participating in this study provided written informed consent before blood collection.

The total RNA was extracted from blood samples with an RNAprep pure Blood Kit (TIANGEN, Beijing, China). Total RNA was quantified with a NanoDrop spectrophotometer 2000 (Thermo Fisher Scientific, Waltham, MA, USA) and then reversely transcribed into cDNA using random primers with the PrimeScript™ RT Reagent Kit (TaKara, Dalian, China). A quantitative real-time polymerase chain reaction (qRT-PCR) was carried out with TB Green™ Premix Ex Taq™ (TaKara, Dalian, China) on a CFX-96 real-time PCR System (Bio-Rad, Shanghai, China). Expression data was normalized by GAPDH, and the 2^−ΔΔCT^ method was applied to analyze the results. All sequences for RNA primers were purchased from Sangon Biotech, Shanghai, China.

## Results

### Identification of differently expressed genes

**Fig 2:**
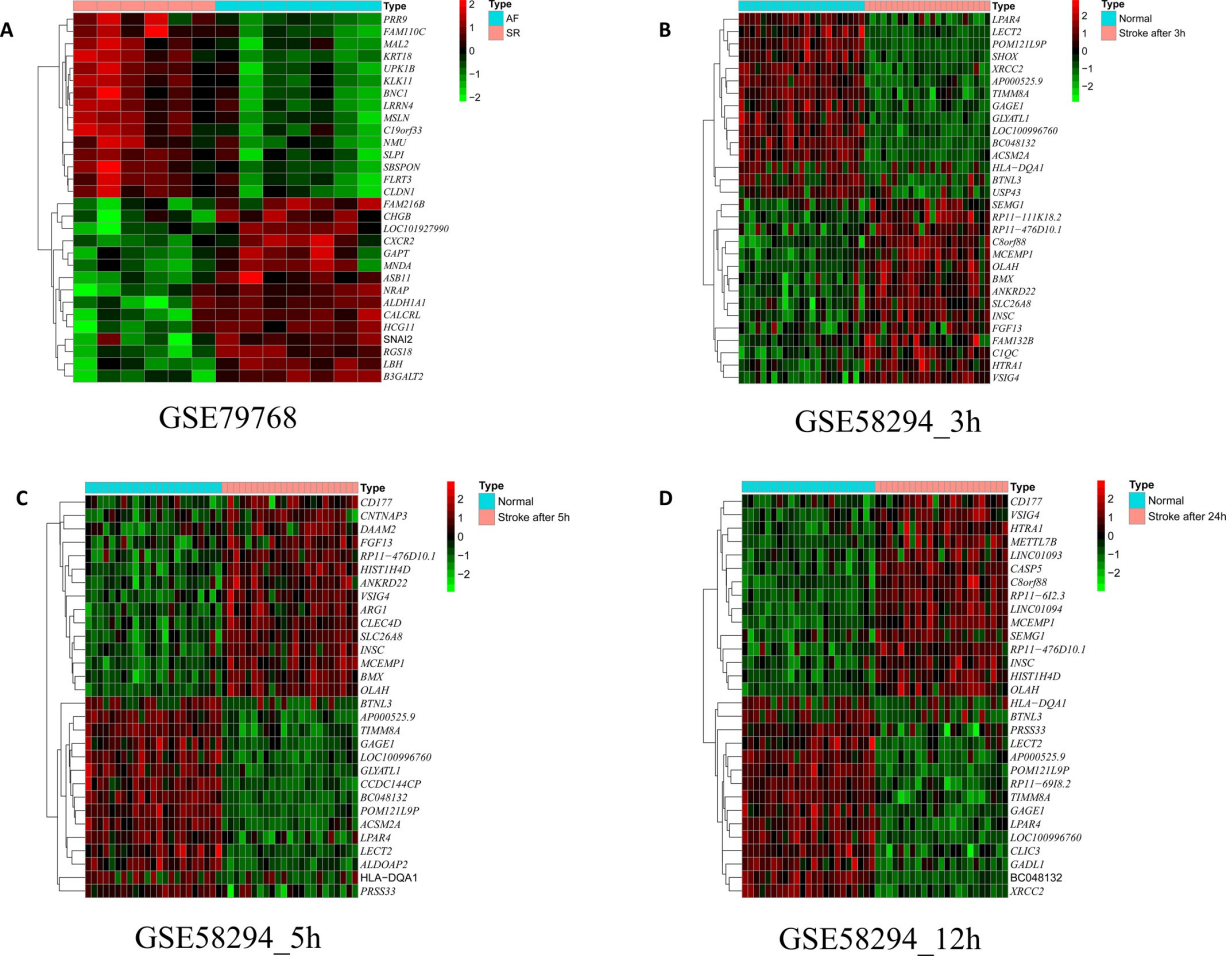
Identification of differentially expressed genes. Heatmap of the top 30 differentially expressed genes based on GSE79768 (A), and at < 3 h (B), 5 h (C), 24 h (D) after stroke in GSE58294. The color intensity (from red to green) suggests the higher to lower expression.

After data pre-processing, we screened the DEGs of the datasets GSE79768, GSE58294, GSE66724, GSE70887, and GSE129409. In GSE79768 (mRNA dataset of AF), we identified 160 upregulated and 93 downregulated genes (**S1 Table**). In GSE58294 (stroke mRNA dataset), we identified 348 upregulated and 445 downregulated genes at <3 h, 555 upregulated and 632 downregulated genes at 5 h, and 525 upregulated and 629 downregulated genes at 24 h. Here, we defined 566 co-expressed DEGs at the three time points mentioned above as the stroke-DEGs (**S2 Table**). In GSE66724 (AF-related stroke mRNA dataset), we identified 195 upregulated and 133 downregulated genes (**S3 Table**). In GSE70887 (AF miRNA dataset), we identified 12 upregulated and 9 downregulated miRNAs (**S4 Table**). In GSE129409 (AF circRNA dataset), we identified 270 upregulated and 162 downregulated circRNAs (**S5 Table**). Heatmaps of the top 30 AF-DEGs and stroke-DEGs are shown in **Fig 2A–2D**.

**Fig 3:**
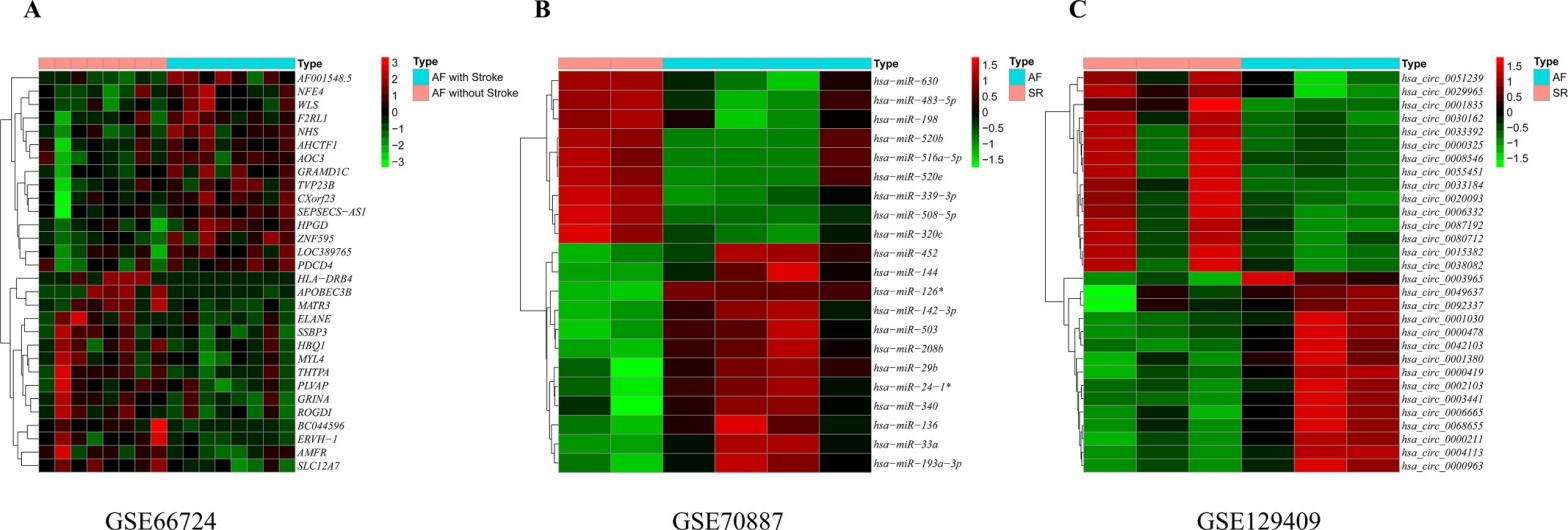
Identification of differentially expressed genes. Heatmap of the top 30 differentially expressed genes based on A GSE66724, B GSE70887, and C GSE129409. The color intensity (from red to green) suggests the higher to lower expression.

The volcanic diagram of all genes and the expression heatmap of the top 30 DEGs in GSE66724 (**S3A Fig, Fig 3A**), GSE70887 (**S3B Fig, Fig 3B**), and GSE129409 (**S3C Fig, Fig 3C**).

### Construction of co-expression networks and identification of key modules

**Fig 4:**
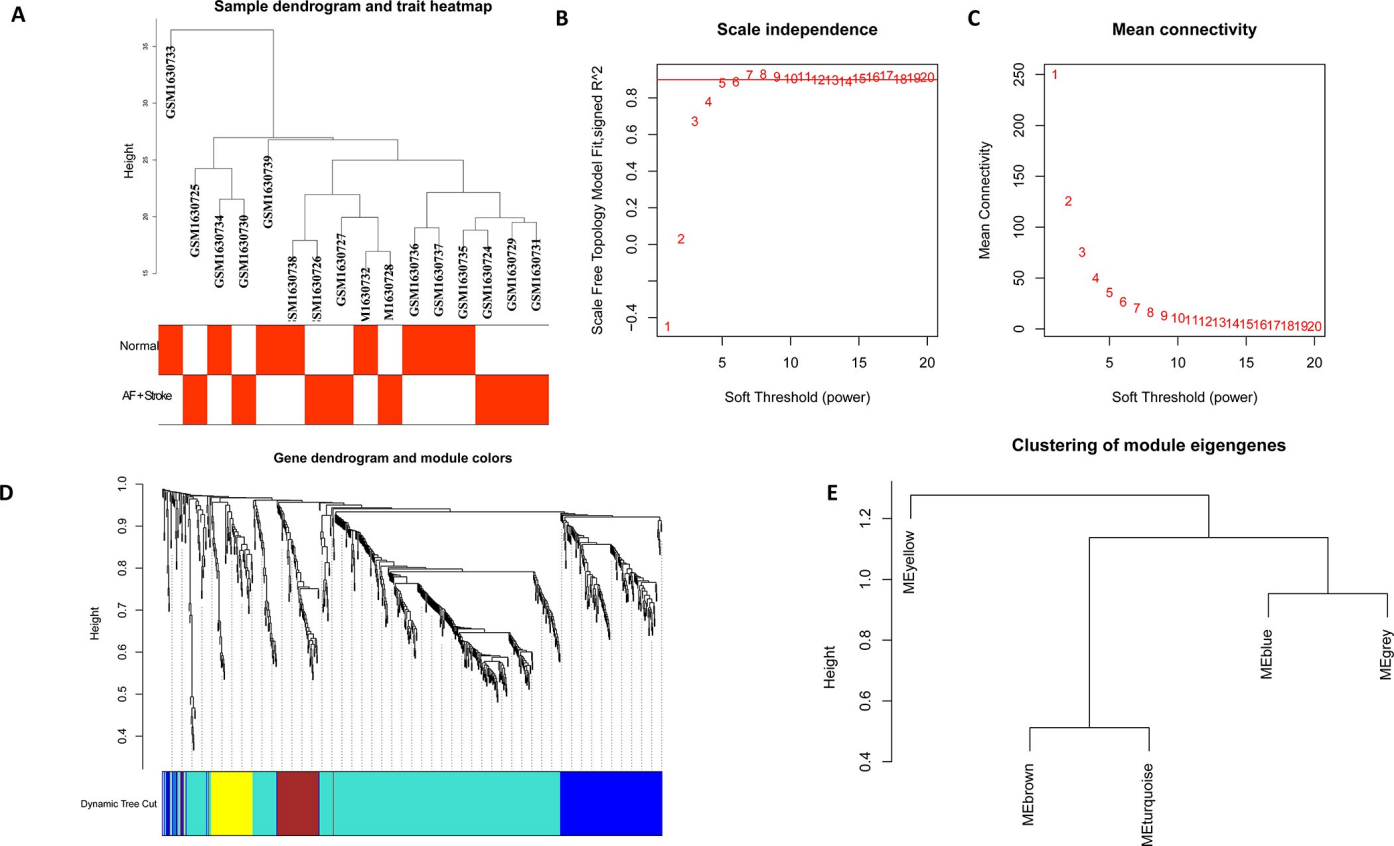
Sample clustering and network construction of the weighted co-expressed genes. Clustering dendrogram of 8 AF without Stroke and 8 AF with Stroke (A). The color intensity was proportional to disease status (with or without Stroke). Analysis of the scale independence (B) and the mean connectivity (C) for various soft‑thresholding powers. The soft‑thresholding power of 5 was selected based on the scale‑free topology criterion. Dendrogram clustered was based on a dissimilarity measure (1‑TOM). Gene expression similarity is assessed by a pair‑wise weighted correlation metric and clustered based on a topological overlap metric into modules. Each color below represents one co‑expression module, and every branch stands for one gene (D). The cluster dendrogram of module eigengenes was demonstrated (E).

**Fig 5:**
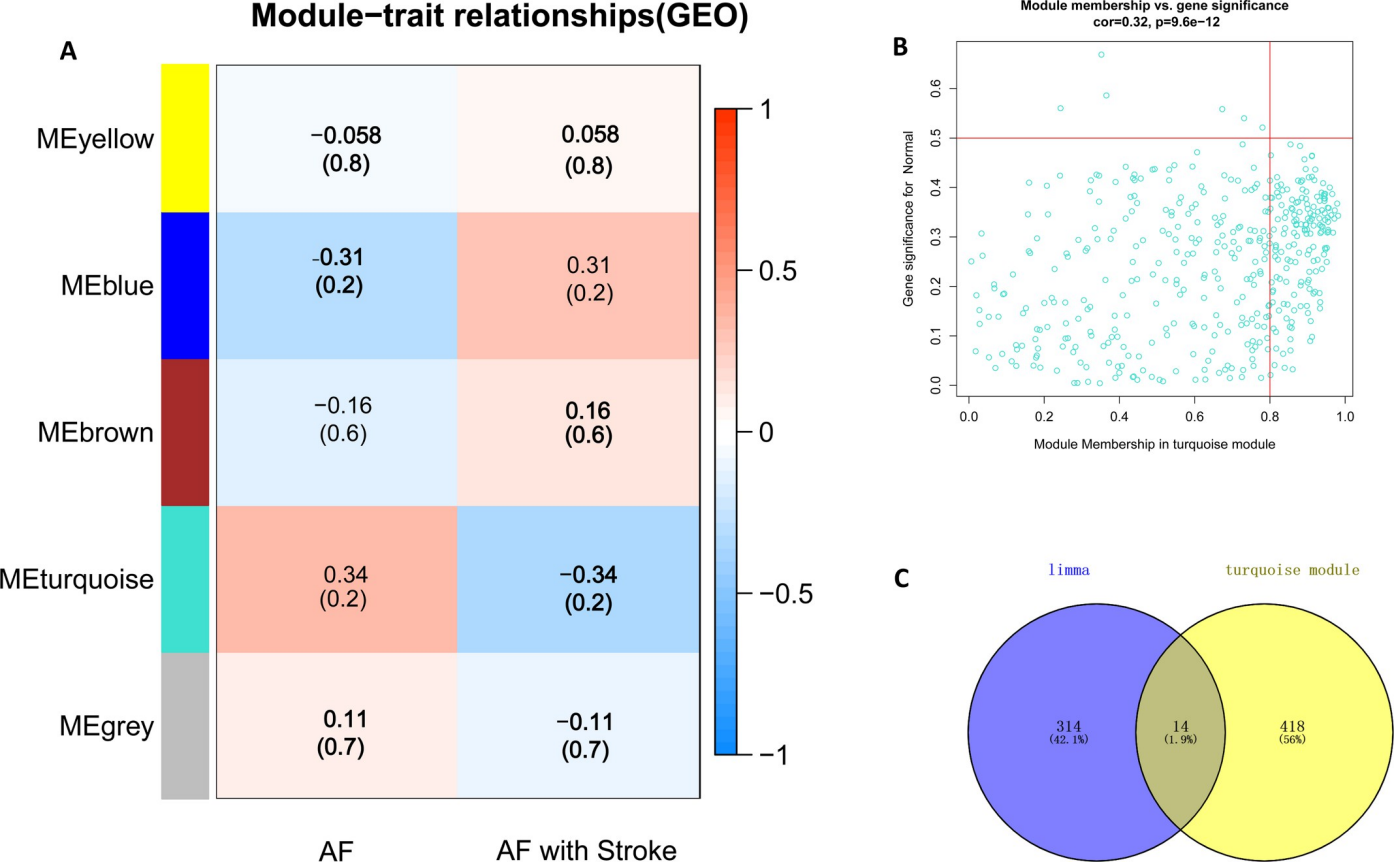
The identification of key modules via weighted gene co‑expression network analysis. Heatmap of the correlation between module eigengenes and the disease status of AF-related Stroke (A). The corresponding correlation coefficient along with P‑value is given in each cell, and each cell is color‑coded by correlation according to the color (legend at right). The turquoise module was most significantly correlated with AF-related Stroke. Scatter plot of module eigengenes in the turquoise module was presented (B). The Venn diagram of genes from the key module and DEGs from GSE66724 was drawn (C).

A hierarchical clustering tree was created based on the dynamic hybrid cut with a scale-free network and topological overlaps (**Fig 4A**). Based on the scale-free topology criterion, a soft-thresholding power of 5 was selected (scale-free R^2^ = 0.85; **Fig 4B and 4C**). Five modules were identified for further analysis. The cluster dendrogram of the modules is shown in **Fig 4D**, while the clustering of module eigengenes is provided in **Fig 4E**. Moreover, we analyzed the association of gene modules by comparing AF with stroke and AF without stroke (**Fig 5A)**. The turquoise module showed the highest positive correlation (r = 0.34). Therefore, we identified the turquoise module as the key one for further analysis. A total of 432 genes were included in the turquoise module (**S6 Table**). Moreover, we illustrated in turquoise the module membership and gene significance for AF with stroke (correlation coefficient = 0.32, *P < 0*.*001*) (**Fig 5B**). In addition, genes in the turquoise module were overlapped with DEGs in GSE66724. Fourteen genes (DEG2), including *ZNF595*, *BC044596*, *MATR3*, *LOC389765*, *PDCD4*, *SEPSECS-AS1*, *GRINA*, *ELANE*, *TVP23B*, *AMFR*, *ROGDI*, *ZNF700*, *PLVAP*, *and MYL4*, were hub gene candidates. We overlapped these two gene sets (DEG3) and obtained 21 genes: *ZNF595*, *MATR3*, *PDCD4*, *SEPSECS-AS1*, *GRINA*, *ELANE*, *TVP23B*, *AMFR*, *ROGDI*, *ZNF700*, *PLVAP*, *MYL4*, *RNF166*, *ACKR4*, *EIF4E3*, *PDZK1IP1*, *HTRA1*, *NELL2*, *BEX2*, *ANXA3*, and *SLC51A*.

### PPI network analysis and functional GO term enrichment analysis

**Fig 6:**
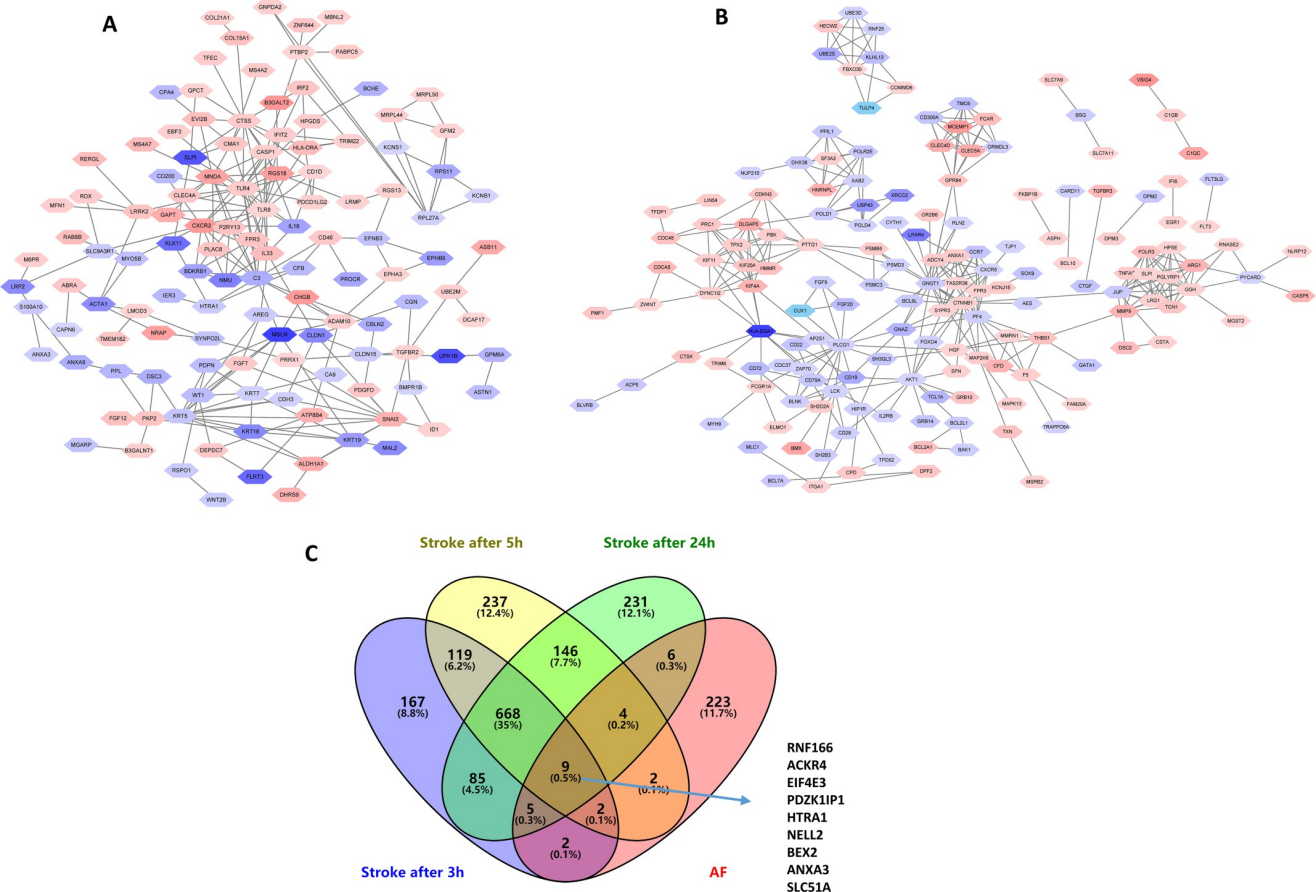
PPI network of AF-related DEGs (A), PPI network of Stroke-related DEGs (B), and Venn diagrams of AF-related stroke genes (C) were presented. Red, greater degree. blue, lesser degree.

We identified 155 and 43 nodes from the PPI network of AF-DEGs and stroke-DEGs, as shown in **Fig 6A and 6B**. The top 10 hub nodes in AF-DEGs included Toll-like receptor 4 (*TLR4*, degree = 18), Toll-like receptor 8 (*TLR8*, degree = 16), complement C3 (*C3*, degree = 15), cathepsin S (*CXCR2*, degree = 13), keratin 5 (*KRT5*, degree = 12), myeloid cell nuclear differentiation antigen (*MNDA*, degree = 11), snail family transcriptional repressor 2 (*SNAI2*, degree = 11), caspase 1 (*CASP1*, degree = 9), and purinergic receptor P2Y13 (*P2RY13*, degree = 9). The top 10 hub genes in stroke-DEGs included G protein subunit gamma transducin 1 (*GNGT1*, degree = 18), phospholipase C gamma 1 (*PLCG1*, degree = 15), platelet factor 4 (*PF4*, degree = 15), AKT serine/threonine kinase 1 (*AKT1*, degree = 14), gamma-glutamyl hydrolase (*GGH*, degree = 13), catenin beta 1 (*CTNNB1*, degree = 13), junction plakoglobin (*JUP*, degree = 11), major histocompatibility complex, class II, DQ alpha 1 (*HLA-DQA1*, degree = 11), LCK proto-oncogene (*LCK*, degree = 11), and adenylate cyclase 4 (*ADCY4*, degree = 11). **Fig 6C** showed that DEG1 owned 9 genes, including *RNF166*, *ACKR4*, *EIF4E3*, *PDZK1IP1*, *HTRA1*, *NELL2*, *BEX2*, *ANXA3*, *SLC51A*.

**Fig 7:**
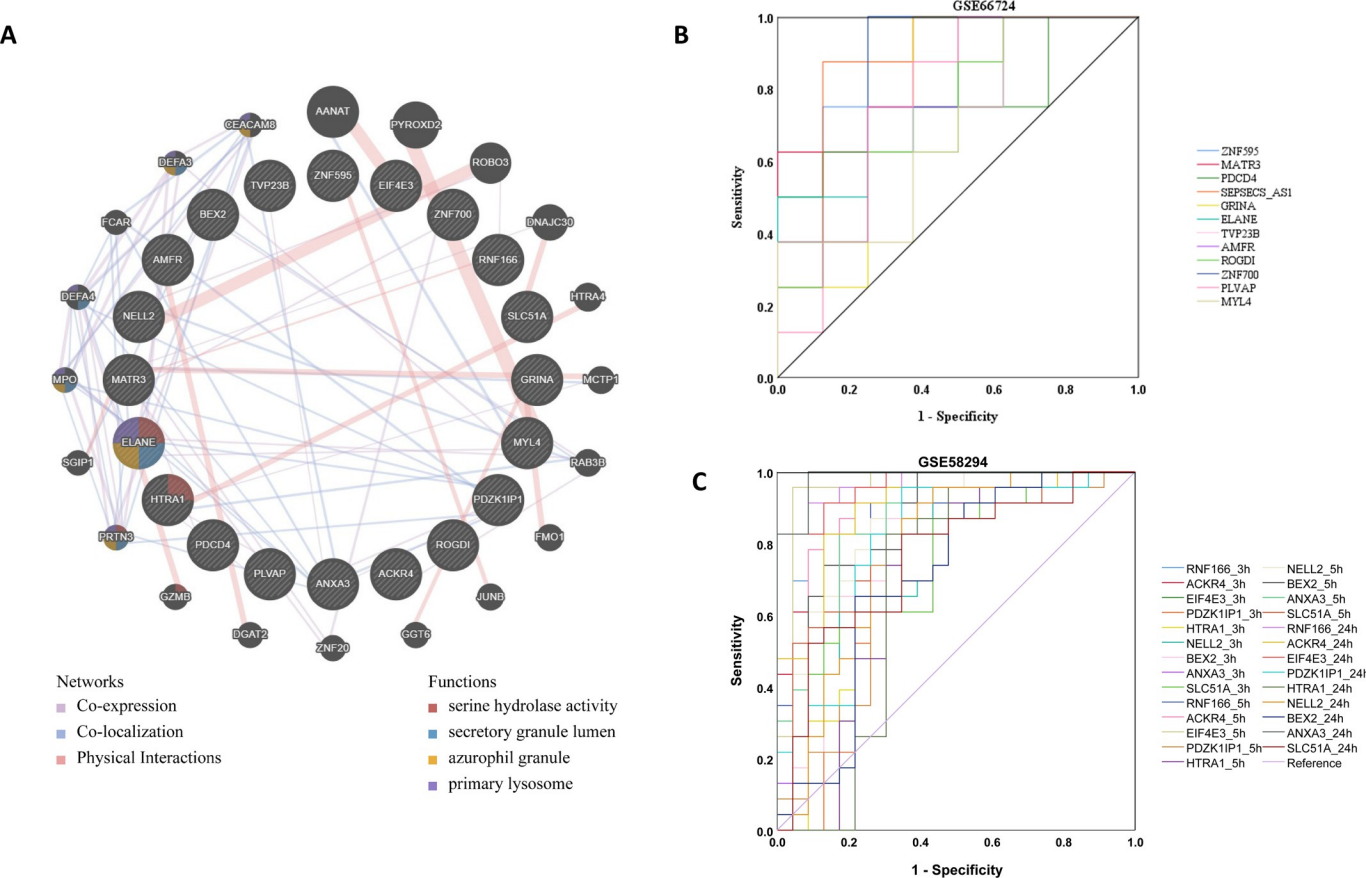
Enrichment analysis of key modules. Gene ontology enrichment analysis in DEG1 (A), DEG2 (B), and DEG3 (C). The significance of enrichment gradually increases from blue to red, and the size of the dots indicates the number of genes contained in the corresponding pathway. ROC curves of hub genes in GSE66724 (D) and GSE58294 (E) were present.

We performed interactomics and GO functional enrichment analysis on DEG3 based on GeneMania (http://genemania.org/). As shown in **Fig 7A**, hydrolase activity, secretory granule lumen, azurophil granule and primary lysosome were the main enriched terms.

### Validation of hub genes of AF-related stroke

**Fig 8:**
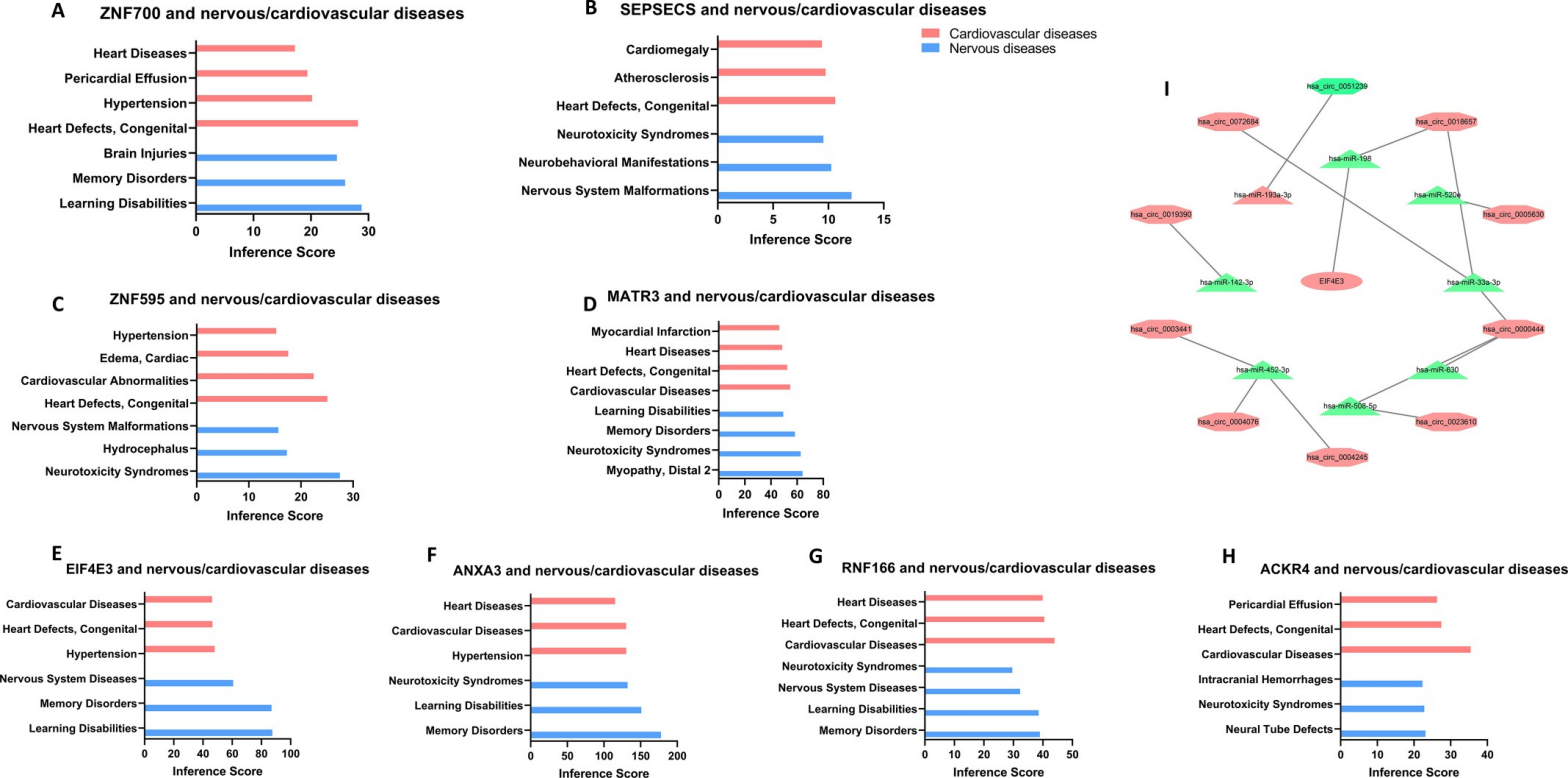
Nervous and cardiovascular diseases related to hub genes based on the CTD database (A-H), circRNA-miRNA-mRNA network (I).

Based on the results of DEG3 and ROC curves in GSE66724 and GSE58294 (**Fig 7D and 7E**), we filtered eight hub genes of AF-related stroke (AUC > 0.8), including *EIF4E3*, *ZNF595*, *ZNF700*, *MATR3*, *ACKR4*, *ANXA3*, *SEPSECS-AS1*, and *RNF166*. The database showed that hub genes targeted several nervous system and cardiovascular diseases (**Fig 8A–8H)**. GO term enrichment related to biological processes, molecular functions, and cellular components of hub genes were associated with various processes, as indicated in **Table 1**.

**Table 1:** The Gene Ontology (GO) terms enrichment for hub genes of the AF-related stroke.

Gene	GO class (direct)	Reference
ZNF595	protein binding	PMID:32296183
	metal ion binding	GO_REF:0000043
	regulation transcription by RNA polymerase l	PMID:21873635
	DNA-binding transcription factor activity, RNA polymerase ll-specific	PMID:21873635
SEPSECS-AS1	tRNA binding	GO_REF:0000024
	protein binding	PMID:22190034
	selenocysteine incorporation	PMID:21873635
	tRNA binding	PMID:21873635
	DNA-binding transcription factor activity, RNA polymerase ll-specific	PMID.21873635
ZNF700	nucleus	PMID:.21873635
	regulation of transcription by RNA polymerase ll RNA polymerase lI transcription regulatory region sequence-specific DNA binding	PMID-21873635
RNF166	protein binding	PMID:32814053
	metal ion binding	GO_REF:0000043
	autophagy	GO_REF:0000043
	innate immune response	GO_REF:0000043
	cytoplasm	GO_REF:0000044
ACKR4	chemokine receptor activity	PMID:23341447
	scavenger receptor activity	GOREF:0000002
	protein binding	PMID:23341447
	chemokine-mediated signaling pathway	GO_REF:0000108
EIF4E3	early endosome	GO_REF:0000044
	mRNA cap binding complex	GO_REF:0000024
MATR3	heart valve development ventricular septum development	GO_REF:00000242
	RNA binding	PMID:226586742
	structural molecule activity	PMID:226818892
	protein binding	PMID:20330752
ANXA3	cytoplasm	PMID:21873635
	calcium ion binding	PMID:21873635

### Reconstruction of the circRNA-miRNA-mRNA network in AF-related stroke

As shown in **Fig 8I**, a circRNA-miRNA-mRNA regulatory network bearing ten circRNAs, eight miRNAs, and one mRNA was constructed to demonstrate pathophysiologic mechanisms in AF-related stroke. The parameters of degree, closeness, and betweenness in the network were calculated by the plugin cytoHubba in Cytoscape. The top 5 nodes were *hsa-miR-33a-3p*, *hsa_circ_0000444*, *hsa-miR-452-3p*, *hsa-miR-198*, and *hsa_circ_0018657*. *EIF4E3* is the target of *hsa-miR-198*, and *hsa-miR-198* is the target of *hsa_circ_0018657*, suggesting that *hsa_circ_0018657/hsa-miR-198/EIF4E3* could be an important pathway regulating the development of AF-related stroke.

### Experimental validation of hub genes

**Fig 9:**
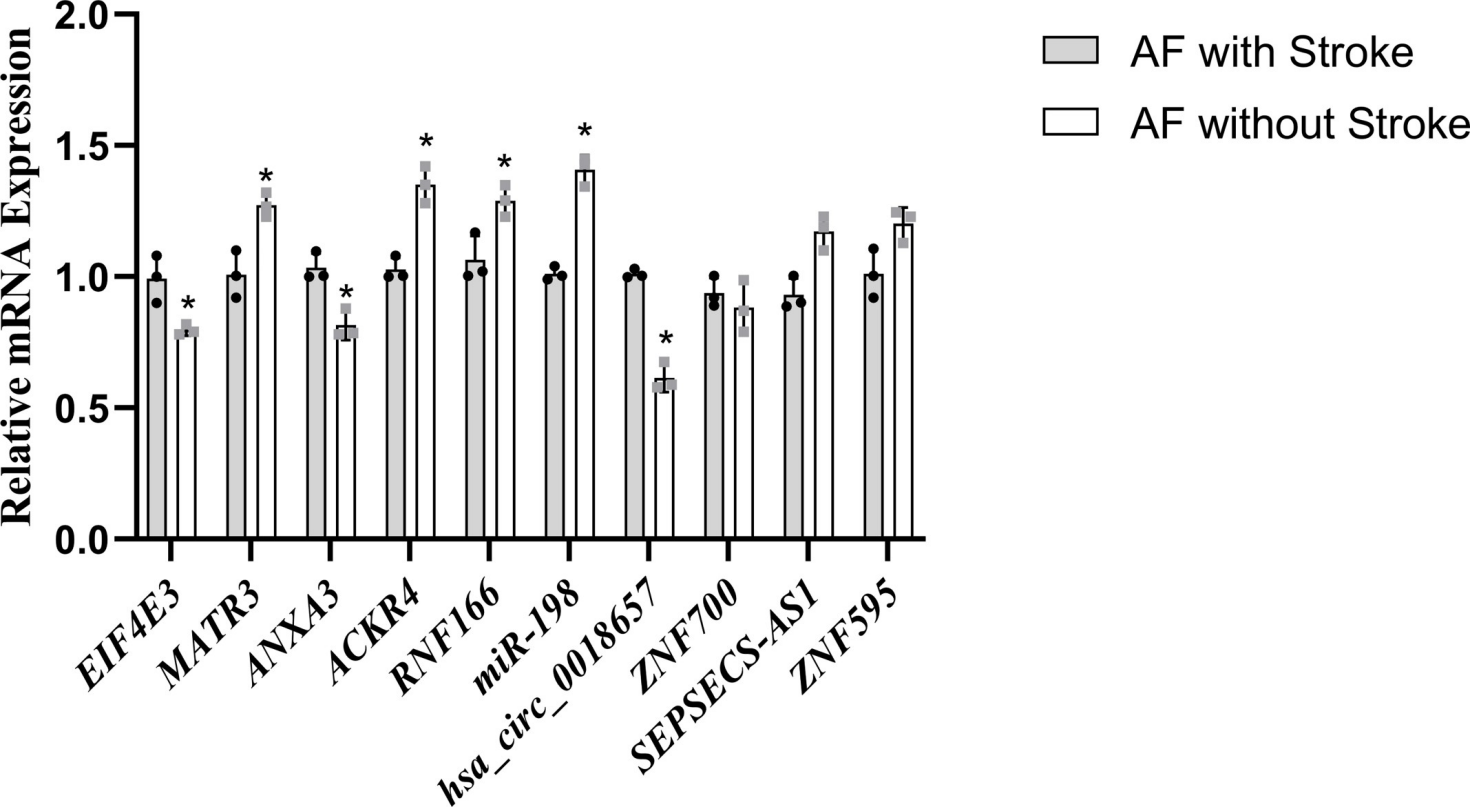
The expression levels of 8 hub genes, miR-198, and hsa_circ_0018657 (n = 3). *: *P < 0*.*05*.

The expression levels of eight hub genes, *miR-198*, and *hsa_circ_0018657* were detected in blood samples by qRT-PCR (**Fig 9A–9J**). Results showed that the expression of *EIF4E3*, *ANXA3*, and *hsa_circ_0018657* was significantly higher in AF-related stroke patients than those in AF patients without stroke, which was consistent with the bioinformatic analysis. The expression of *MATR4*, *ACKR4*, *RNF166*, and *miR-198* in AF-related stroke patients was lower than those in AF patients without stroke.

## Discussion

As a serious public health concern, AF has shown an increasing incidence and prevalence in the elderly population, which is associated with elevated risks of cerebrovascular disease events and deaths [20–22]. Discriminating individuals with AF who are prone to develop atrial mural thrombus and cardiogenic stroke is of great concern. Despite continuous cardiac rhythm monitoring, around 30% of patients show no obvious signs before the occurrence of cerebrovascular events. Thus, identifying biomarkers of AF-related stroke and elucidating the relationship between AF and embolic events may provide novel therapeutic targets for primary care [23].

In this study, a series of bioinformatics analyses were carried out to filter hub genes of AF-related stroke. We first took the overlap of DEGs from AF and stroke patients to obtain DEG1. Then, WGCNA was used to identify key modules associated with AF-related stroke to obtain DEG2. The overlap of DEG1 and DEG2 (DEG3) was considered to include important genes in the development of AF-related stroke. Then, the PPI network of AF- and stroke-related DEGs was constructed, and we filtered eight hub genes based on ROC analyses. Those hub genes could facilitate the prevention of AF-related stroke and provide novel therapeutic targets. We also investigated the biological processes, cellular components, and molecular functions of the DEGs, revealing that these genes are significantly associated with the regulation of the BMP signaling pathway, smooth muscle cell proliferation, and extracellular matrix disassembly. Finally, we constructed a circRNA-miRNA-mRNA network of AF-related stroke and screened out the *hsa_circ_0018657/hsa-miR-198/EIF4E3* axis as an important regulatory pathway in the development of AF-related stroke. These results were further validated in our qRT-PCR experiments.

In the present study, *EIF4E3* was explored as a potential molecular signature for AF patients with a high probability of stroke occurrence, which may play an important role in the development of AF-related stroke. *IF4E3* belongs to the *EIF4E* family as a translational initiation factor that interacts with the 5-prime cap structure of mRNA and recruit mRNA to the ribosome. Interestingly, although considered to regulate the nervous system directly or indirectly, *EIF4E3* also has a role in the cardiovascular system. Mrvová, S and colleagues performed bioinformatics analyses of expressed sequence tags and the 3’-UTRs of the main transcript splice variants of the translational initiation factor *EIF4E3* and showed that *EIF4E3* mRNAs have a great potential in heavy post-transcriptional regulation [24]. *EIF4E3* truncated transcript variants were mainly found in the brain. However, *EIF4E3* also promotes angiogenesis in the region surrounding myocardial infarction [25].

Along with genetic susceptibility, thrombus vulnerability is another main reason for AF-related stroke. miRNAs have also been proposed as potential biomarkers for vulnerable plaques. Hoekstra, M *et al*. explored peripheral blood mononuclear cell microRNA profiles from coronary artery disease patients and found that *miR-198* exhibited a high expression level in unstable angina pectoris patients compared with such levels in stable patients [26]. Sepramaniam, S and colleagues performed a miRNA microarray of peripheral blood samples from acute stroke patients and healthy controls, presenting that miR-198 was dysregulated in stroke patients [27], which is consistent with our results. Based on previous studies, we believe that *miR-198* could play an important role in the development of AF-related stroke by binding to the 3’UTR of EIF4E3.

As the host gene of *hsa_circ_0018657*, *EIF4EBP2* was also found to participate in brain dysfunction. Martín-Flores, N showed the significant association of SNP rs1043098 in the *EIF4EBP2* gene with the onset of dyskinesia induced by L-DOPA administration [28]. In multiple sclerosis patients, *EIF4EBP2* expression was downregulated compared to those in healthy controls [29]. Thus, *hsa_circ_0018657* could also play a part in cerebral disease, which needs further verification. As a miRNA sponge, *hsa_circ_0018657* could regulate *miRNA-198* by targeting *EIF4E3*. The role of this axis in the development of AF-related stroke deserves further exploration.

*ZNF595* and *ZNF700* belong to the zinc finger protein family, whose members function as transcription factors that can regulate a broad variety of developmental and cellular processes. In schizophrenia (SCZ) patients, nonsense *de novo* mutations of *ZNF595* are common. Data from genome-wide association studies suggested that common variants in the *ZNF595* gene may be associated with SCZ and SCZ-related traits [30]. Pathogenic mutations in selenocysteine synthase (SEPSECS) cause neurodevelopmental disorders [31–33]. While using sequencing data analysis, Laan, L identified regional epigenetic changes in the transcription factor gene *ZNF700*, which is relevant in Down syndrome brain development, providing a novel framework for further studies on epigenetic changes and transcriptional dysregulation during chromosome 21 neurogenesis [34].

The *MATR3* gene encodes a nuclear matrix protein, which is proposed to stabilize certain mRNA species. Previous studies proved that mutations in *MATR3* cause hereditary amyotrophic lateral sclerosis [35–40]. In a genome-wide association study using memory performance in a cohort of elderly individuals (>60years), *MATR3* was significantly associated with neuronal development, synaptic plasticity, and memory-related processes [41]. In addition, the 3’ UTR of *MATR3* encodes the nuclear matrix protein *MATR3*, which is strongly expressed in the neural crest, developing heart, and great vessels [42]. Thus, subtle perturbations in *MATR3* expression appear to cause similar left ventricular outflow tract defects in humans and mice. *ACRK4* is a member of the G protein-coupled receptor family and is a receptor for C-C type chemokines. *ACKR4* binds the homeostatic chemokines *CCL19*, *CCL21*, *CCL25*, and *CXCL13* and has been attributed scavenging properties [43]. The expression of *ACKR4* was upregulated in the border/infarct area after myocardial infarction, and knocking out *ACKR4* protected against adverse ventricular remodeling in mice post-infarction, indicating that *ACKR4* may be a novel therapeutic target to ameliorate cardiac remodeling [44]. *ANXA3* is recognized as a regulator of cerebral ischemia/reperfusion injury. The upregulation of *ANXA3* could promote cell viability, decrease cell apoptosis, and reduce the production of inflammatory cytokines in neurons after oxygen-glucose deprivation [45]. The silencing of *ANXA3* would promote repair and healing of the myocardium after infarction by the activation of the PI3K/Akt signaling pathway [46]. Thus, there may be a relationship between cardiovascular and nervous system diseases, arising from loci mutations or gene variants [23].

This study also has several limitations. First, this study was based on microarray analysis, and gene expression may be not directly equivalent to protein expression. Second, data for the analysis of AF-related stroke mainly refers to persistent AF patients. Although persistent AF was most hazardous in stroke cases, other AF forms should be studied in the future. Finally, although we performed qRT-PCR to verify the expression levels of genes, more *in vitro* and *in vivo* experiments should be carried out to validate our results.

## Conclusion

The hub genes of *EIF4E3*, *ZNF595*, *ZNF700*, *MATR3*, *ACKR4*, *ANXA3*, *SEPSECS-AS1*, and *RNF166* may link AF and secondary stroke. The *hsa_circ_0018657/hsa-miR-198/EIF4E3* pathway could be an important regulating axis in AF-related stroke. In addition, the hub genes *TLR4*, *TLR8*, *C3*, *CXCR2*, *KRT5*, *MNDA*, *SNAI2*, *CASP1*, *and P2RY13* may be associated with AF recurrence and maintenance. *GNGT1*, *PLCG1*, *PF4*, *AKT1*, *GGH*, *CTNNB1*, *JUP*, *HLA-DQA1*, *LCK*, *and ADCY4* may be associated with stroke.

## Supporting information

S1 Fig The venn plot of these three DEG datasets. (TIF)

S2 Fig The PPI network of DEG3. (TIF)

S3 Fig The volcanic diagram of all genes in GSE66724. (TIF)

S1 Table 160 upregulated and 93 downregulated mRNAs in GSE79768. (XLSX)

S2 Table 583 co-expressed DEGs at the three time points in GSE58294. (XLSX)

S3 Table 195 upregulated and 133 downregulated genes in GSE66724. (XLSX)

S4 Table 12 upregulated and 9 downregulated genes in GSE70887. (XLSX)

S5 Table 270 upregulated and 162 downregulated genes in GSE129409. (XLSX)

S6 Table 432 genes in the turquoise module. (XLSX)
