# Supplementary figures and images for: Integrative identification of hub genes in development of atrial fibrillation related stroke
Source: PLoS One. 2023 Mar 23;18(3):e0283617. doi: 10.1371/journal.pone.0283617 (PMC10035830; doi:10.1371/journal.pone.0283617)

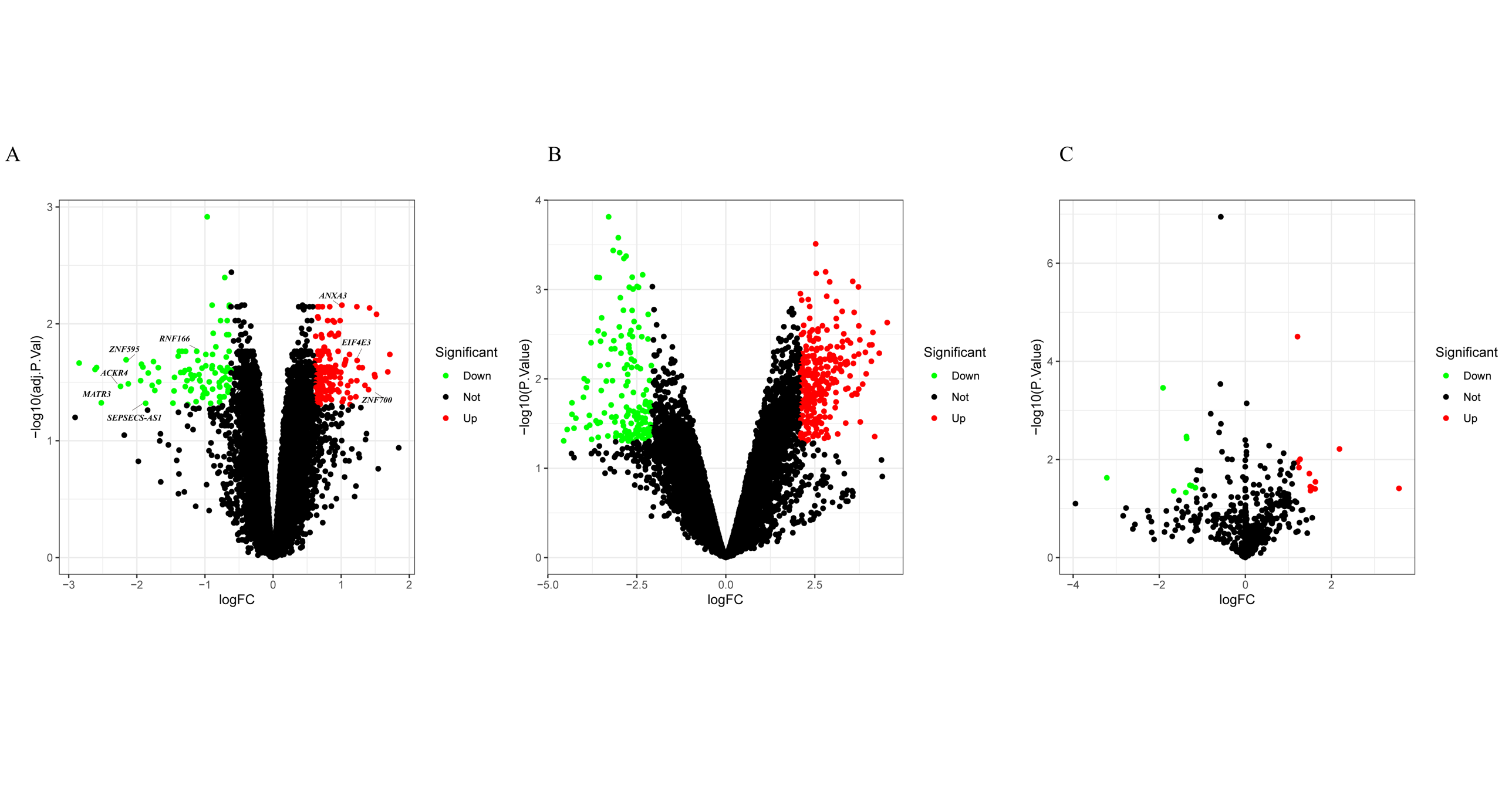

Supplement: S1 Fig — (TIF) [file pone.0283617.s001.tif]

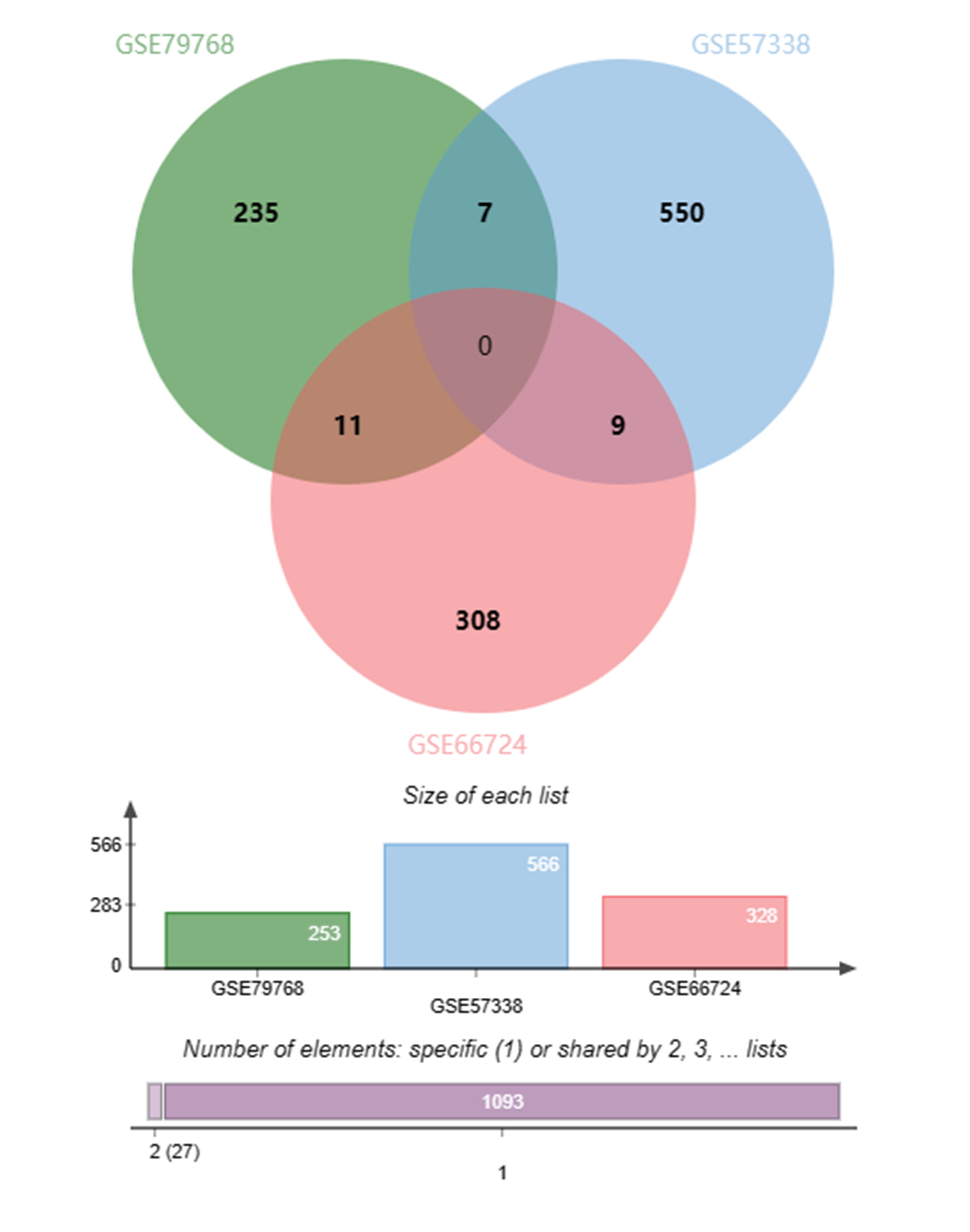

Supplement: S2 Fig — (TIF) [file pone.0283617.s002.tif]

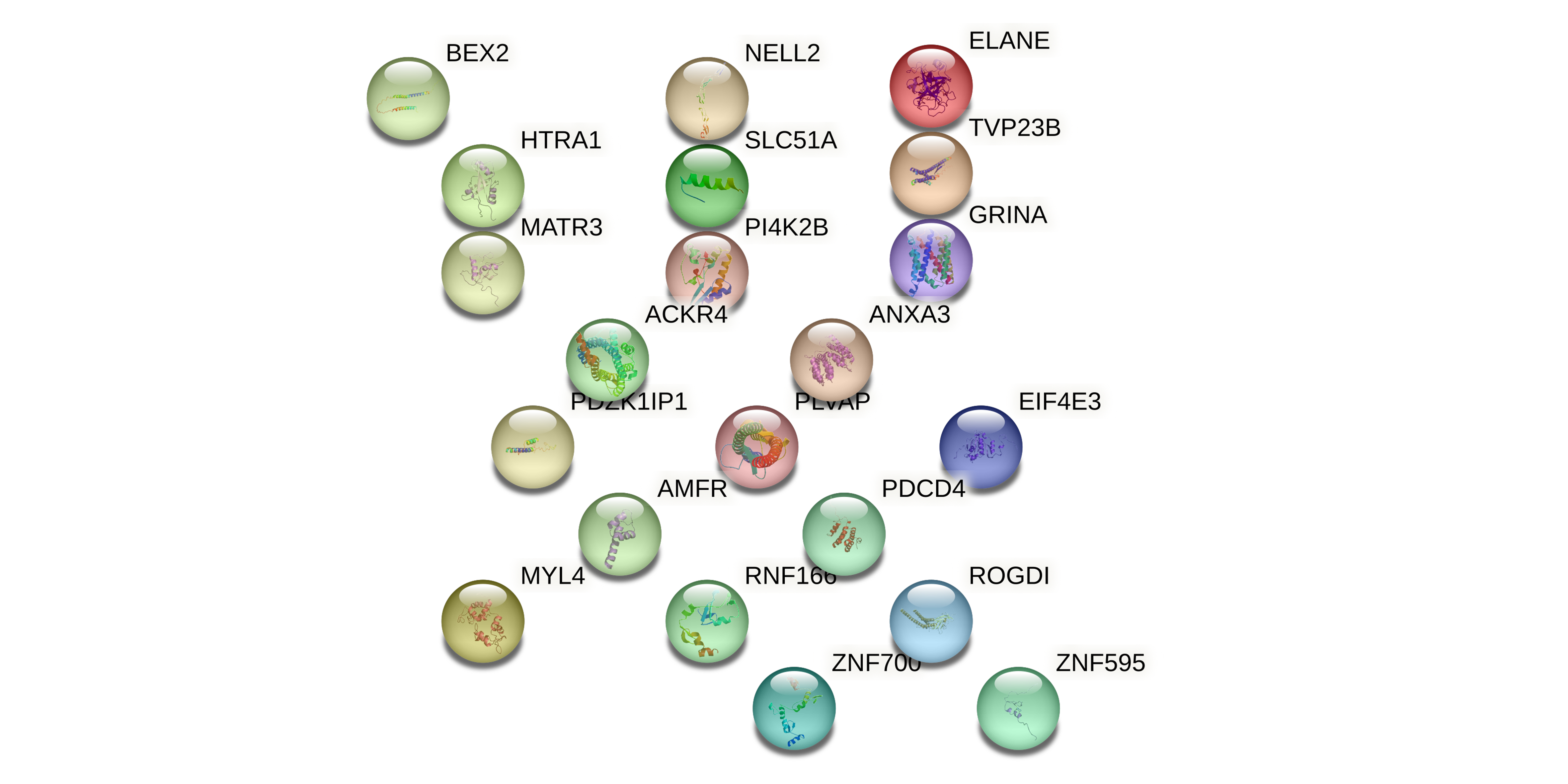

Supplement: S3 Fig — (TIF) [file pone.0283617.s003.tif]
